# A Comparative Evaluation of Antibacterial Efficacy of *Moringa oleifera* Leaf Extract, Octenidine Dihydrochloride, and Sodium Hypochlorite as Intracanal Irrigants against *Enterococcus faecalis*: An In Vitro Study

**DOI:** 10.1155/2023/7690497

**Published:** 2023-03-14

**Authors:** Afrah M. Alharbi, Tahani M. Alharbi, Mashael S. Alqahtani, Fathy M. Elfasakhany, Ibtesam K. Afifi, Mona T. Rajeh, Mohamed Fattouh, Laila Mohamed Mohamed Kenawi

**Affiliations:** ^1^Bachelor of Dental Medicine and Surgery, Faculty of Dental Medicine, Umm Al-Qura University, Makkah, Saudi Arabia; ^2^Department of Basic and Clinical Oral Sciences, Faculty of Dental Medicine, Umm Al-Qura University, Makkah, Saudi Arabia; ^3^Department of Medical Biochemistry, Faculty of Medicine, Tanta University, Tanta, Egypt; ^4^Department of Medical Microbiology and Immunology, Faculty of Medicine, Tanta University, Tanta, Egypt; ^5^Department of Dental Public Health, Faculty of Dentistry, King Abdulaziz University, Jeddah, Saudi Arabia; ^6^Department of Fixed Prosthodontics, Faculty of Dentistry, Cairo University, Cairo, Egypt; ^7^Department of Oral and Maxillofacial Surgery, Faculty of Dental Medicine, Umm Al-Qura University, Makkah, Saudi Arabia; ^8^Department of Endodontics, Faculty of Dentistry, Cairo University, Cairo, Egypt; ^9^Department of Restorative Dentistry, Faculty of Dental Medicine, Umm Al-Qura University, Makkah, Saudi Arabia

## Abstract

**Objectives:**

The aim of this study is to investigate and compare the microbial efficacy of *Moringa oleifera* leaf extract, octenidine dihydrochloride (OCT), NaOCl, and their combinations as intracanal irrigants against *Enterococcus faecalis*.

**Materials and Methods:**

Sixty single-rooted mandibular premolars were decoronated followed by root canal preparation. Each root specimen was autoclaved, inoculated with *E. faecalis*, and incubated at 37°C for 48 hr. Then, the specimens were divided into six groups based on the irrigation solution used: 2.5% NaOCl (Group 1), 0.1% OCT (Group 2), *M. oleifera* leaves extract (Group 3), a combination of *M. oleifera* extract and 1.25% NaOCl (Group 4), a combination of *M. oleifera* extract and OCT (Group 5) and normal saline (Group 6). Microbial samples were taken from each root canal before (S1) and after (S2) irrigation and the bacterial viability was assessed using colony-forming units (CFU) on bile esculin agar plates.

**Results:**

Comparing the number of CFU/ml before and after irrigation showed a significant reduction (*P* < 0.001) in all studied groups. Comparison between the CFU/ml after irrigation by NaOCl and each of the combination groups showed a significant difference.

**Conclusion:**

*M. oleifera* leaves extract and 0.1% OCT solutions have antibacterial effect against *E. faecalis* comparable to 2.5% NaOCl and might be used as root canal irrigants. The combination groups showed better antimicrobial activities than individual irrigants. However, further studies are required to investigate the biocompatibility and possible toxic effects of the tested irrigants.

## 1. Introduction

Endodontic therapy aimed to eliminate the microorganisms and their by-products from the root canal system [1]. Although the mechanical instrumentation is one of the main steps in root canal treatment, it cannot achieve the complete removal of microorganisms [2]. Owing to the complex root canal anatomy, antimicrobial irrigation solutions are critical during the cleaning and shaping process. They can penetrate deeply through the dentinal tubules, reach canal ramifications, and other difficult to reach places. Therefore, antimicrobial irrigants have been considered as an adjunct to mechanical instrumentation [3].

*E. faecalis* is a Gram-positive facultative anaerobe that is thought to be the most resistant species in infected root canals and is frequently linked to the failure of endodontic therapy [4], with a prevalence of 29%–77% [5, 6]. Difficulty in the removal of *E. faecalis* from the root canals may be due its inherent antimicrobial resistance as well as its capacity to penetrate deeply into the dentinal tubules and to produce a biofilm [7]. To decrease the endodontic treatment failure rate, intracanal irrigants should be proven effective against *E. faecalis* [8].

Sodium hypochlorite (NaOCl) is the gold standard root canal irrigant and was shown to have strong antimicrobial activity and effective tissue-dissolving property [9–11]. However, its major drawbacks include a cytotoxic effect on the periapical tissues, an inability to remove the smear layer, and unpleasant taste and odor [11, 12]. Thus, an alternative endodontic irrigant with the same efficacy as NaOCl, but less toxic and with better patient acceptance is still being researched.

OCT is an antiseptic used in the medical field [13], owing to its antimicrobial activity against both Gram-positive and Gram-negative bacteria, fungi, yeasts, and some viruses. It has been used as an antiseptic for skin burns, wound disinfection, and mouth rinse. It consists of 0.1% OCT and phenoxyethanol, an ethanol derivative, which serves as a preservative [14, 15]. Numerous studies reported that OCT has a potent antimicrobial effect against *E. faecalis* [16, 17]. Few in vitro studies have demonstrated that OCT has high antimicrobial activity when used as a root canal irrigant [14, 15, 18]. OCT has been considered a substitute for chlorhexidine gluconate (CHX) based on its greater antimicrobial activity, low cytotoxicity, and high biocompatibility [15]. In addition, it has a faster ability to provide intratubular disinfection than NaOCl and CHX [18].

The side effects and persistent increase in the antibiotic-resistant strains have directed researchers toward the use of nontoxic and more biocompatible herbal materials in endodontic therapy [19]. The literature has addressed many natural extracts for endodontic purposes such as *Morinda citrifolia*, Triphala, green tea polyphenols, and neem [20–22]. *M. oleifera* is a native Indian tree belonging to Moringaceae family, commonly known as “drumstick” or “horseradish” tree [23]. It has multiple medicinal uses with high nutritional value [24]. Studies on this plant have revealed that it possesses antibacterial, antiviral, antioxidant, antisclerotic, and antiinflammatory effects. It has been used to treat malnutrition, malaria, colon cancer, and myeloma [25]. The antibacterial activity of *M. oleifera* leaf extract is due to its compounds; saponins, flavonoids, tannins, alkaloids, phenolics, and triterpenoids, which have different mechanisms of killing bacteria [26]. Various studies have reported that *M. oleifera* leaf extract had a significant antimicrobial effect on *E. faecalis* [27–29].

A literature search revealed insufficient evidence for using *M. oleifera* leaf extract or OCT as intracanal irrigants. Moreover, no previous study has compared the antibacterial activity of *M. oleifera* leaf extract, OCT, and NaOCl or used their combinations. Therefore, this study aims to evaluate and compare the antibacterial efficacy of *M. oleifera* leaf extract, OCT, NaOCl, and their combinations as intracanal irrigants against *Enterococcus faecalis*. The null hypothesis in this study was that there would be no difference in the antibacterial efficacy of the tested groups.

## 2. Materials and Methods

This study was carried out in the research laboratory at the Faculty of Dental Medicine, Umm Al-Qura University, Makkah, Saudi Arabia. Ethical approval was obtained from Biomedical Research Ethics Committee, Umm Al-Qura University (Approval No. HAPO-02-K-012-2021-02-575).

### 2.1. Sample Size

The sample size was determined using the statistical power analysis, G^*∗*^ power version 3.1. A power of 95% and a significant level of 0.05 were used. A sample size of 10 single-rooted mandibular premolars per group was established.

### 2.2. Specimen Collection and Preparation

**Figure 1:**
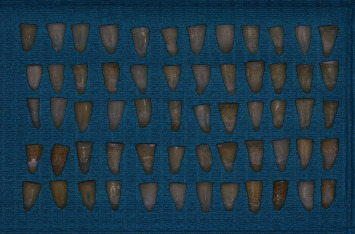
Prepared teeth after decoronation and root canal preparation.

Sixty single-rooted human mandibular premolars with fully developed root apices extracted for periodontal or orthodontic reasons were selected for the study. Exclusion criteria include teeth with previous endodontic therapy, cracks, fracture, curved roots, root caries, and root resorption. Each tooth was radiographed to confirm the presence of a single patent canal. The teeth were cleaned from any calculus or soft tissue debris and then were kept in 0.9% normal saline until instrumentation to avoid dehydration. All teeth were decoronated with a diamond disc under continuous water coolant to obtain a standardized tooth length of ∼14 ± 1 mm and to establish a stable reference point during instrumentation. The working length of each root canal was confirmed by subtracting 1 mm from the length recorded when a tip of a #15 k-file (Dentsply Maillefer, Ballaigues, Switzerland) was visible at the apical foramen. Then, glide path was established for all canals using stainless-steel k-files up to size #25. Canal preparation for all specimens was carried out using ProTaper Next nickel–titanium rotary system (Dentsply Maillefer, Switzerland) till size X4 file. During instrumentation, irrigation was performed with 5 ml of 2.5% NaOCl using a disposable syringe with a 23-gauge needle. The apical foramina were sealed with light-cured composite resin (Figure 1). Each prepared root specimen was autoclaved in a glass tube containing 3 ml of Brain Heart Infusion (BHI) broth at 121°C for 20 min then incubated for 24 hr at 37°C to confirm sterility.

### 2.3. Collection and Processing of Plant Material

Fresh *M. oleifera* leaves were collected from Al-Rajhi plant nursery in Makkah region, Saudi Arabia. The plant was identified by a specialist in Pharmacology and Medicinal Plants, Faculty of Pharmacy, Umm Al-Qura University. The leaves were washed and cleaned thoroughly with tap water followed by sterilized distilled water to eliminate dust and other impurities. The plant leaves were dried at room temperature overnight and then kept in a hot air oven at 45°C for complete removal of moisture. The dried leaves were blended, pulverized, and stored in air-tight containers at 4°C until use. The whole experiment was performed using a single source of *M. oleifera* plant.

### 2.4. Preparation of *M. oleifera* Leaves Extract

**Figure 2:**
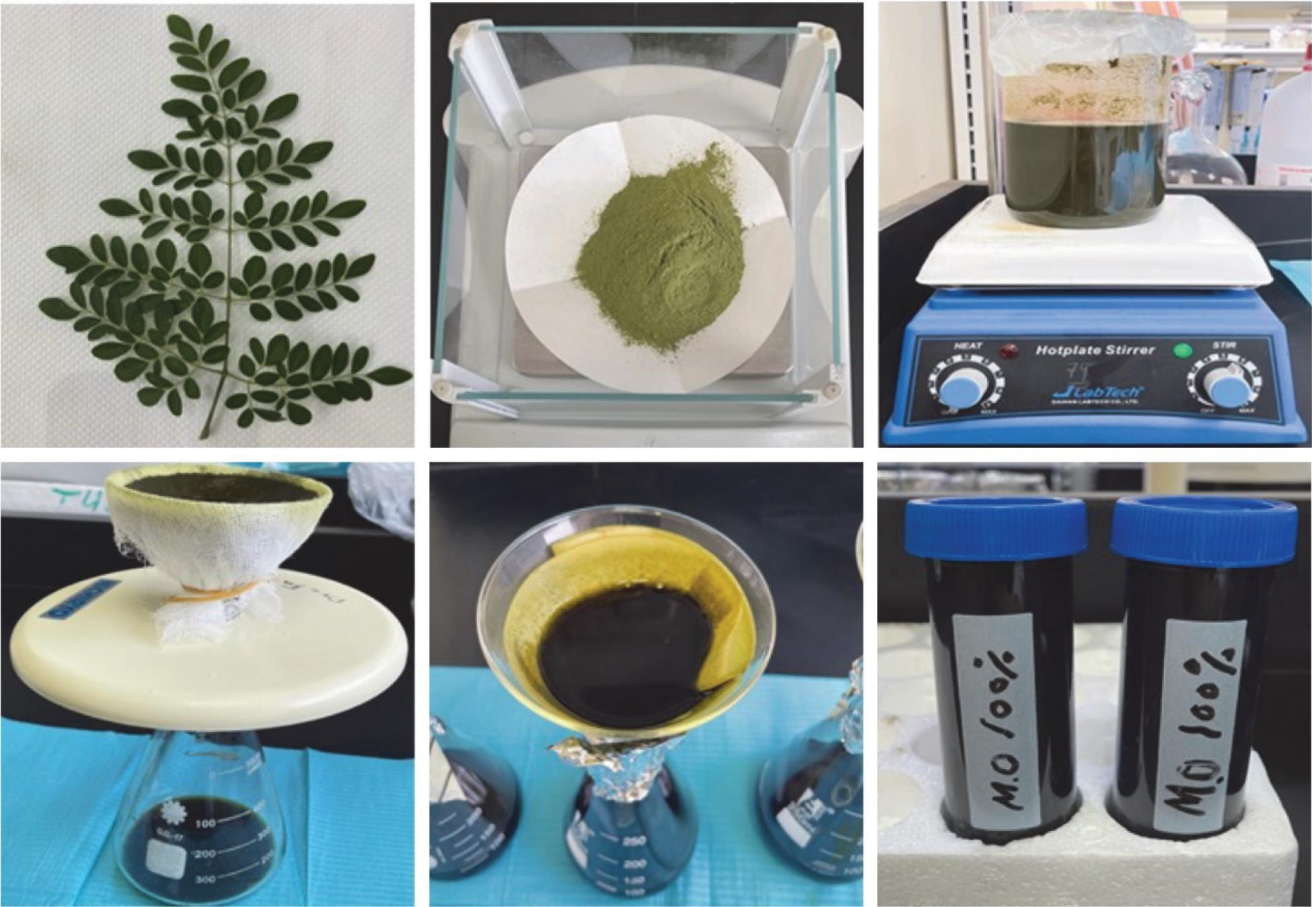
Preparatory procedure of *M. oleifera* leaves extract.

Two hundred grams of *M. oleifera* leaves powder were added to 1,000 ml of 60% ethanol and kept at room temperature for 48 hr. After that, the resulting mixture was magnetically stirred at 800 rpm for 4 hr to achieve a homogenous mixture and then stored at 4°C for 24 hr to allow the extraction of active ingredients. The mixture was filtered through a muslin cloth, then filtered with Whatman No. 1 filter paper. Subsequently, the extract was incubated at 37°C for several days to allow for the evaporation of the solvent to obtain a final volume of 100 ml. The crude extract (100%) was aseptically stored in air-tight containers until use (Figure 2).

### 2.5. Preparation of *E. faecalis* Suspension

**Figure 3:**
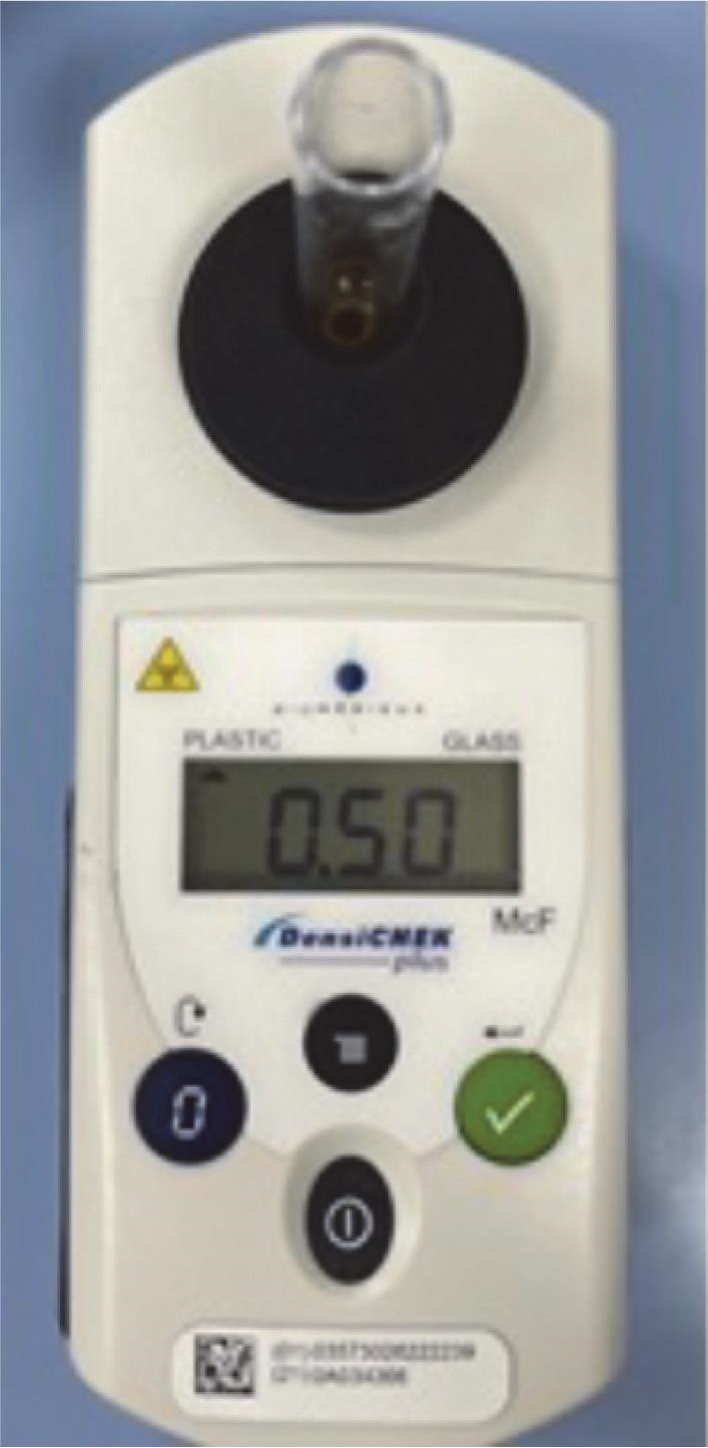
Turbid meter for adjusting turbidity of a bacterial suspension at 0.5 McFarland.

*E. faecalis* (ATCC 29212) strain was inoculated on a bile esculin agar plate at 37°C for 24 hr. Isolated colonies grown on the solid media were suspended in BHI broth and adjusted to the optical density of ∼1.5 × 10^8^ colony forming units (CFU)/ml, by comparing its turbidity to a 0.5 McFarland standard spectrophotometrically (Figure 3).

### 2.6. Inoculation of Root Canals

**Figure 4:**
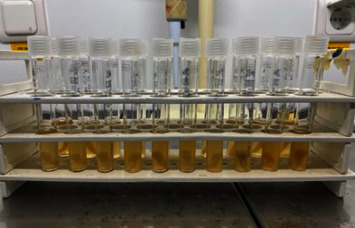
Prepared teeth after incubation.

Two milliliters of sterile BHI broth were removed from the previously autoclaved tubes containing root specimens and replaced by 2 ml of *E. faecalis* suspension. After that, the tubes were sealed and kept at 37°C for 48 hr (Figure 4).

### 2.7. Experimental Groups

The specimens were randomly divided into six groups (*n* = 10 per group) based on the irrigation solution used:

- Group 1: 2.5% NaOCl.
- Group 2: 0.1% OCT (Octenisept; Schülke & Mayr, Norderstedt, Germany).
- Group 3: 100% *M. oleifera* leaves extract.
- Group 4: a combination of 100% *M. oleifera* leaves extract and 1.25% NaOCl.
- Group 5: a combination of 100% *M. oleifera* leaves extract and 0.1% OCT.
- Group 6: 0.9% normal saline (control).

### 2.8. Microbial Sampling and Bacterial Counting

After the incubation period, each root was removed from the tube under complete aseptic conditions and irrigated with 5 ml sterile physiological saline to remove the culture medium and nonadherent bacteria. Bacterial sampling S1 was carried out, just after the incubation period and before using the experimental irrigants, using a dry sterile paper point size 40. The paper point was kept in the root canal for 5 min and then transferred to a sterile test tube containing 1 ml of sterile saline and vortexed for 30 s. Four serial dilutions of each sample were prepared (10^2^, 10^3^, 10^4^, and 10^5^) and used for the bacterial count. After that, aliquots of 10 *µ*l of each dilution were cultured on bile esculin agar plates and incubated at 37°C for 24 hr. The grown *E. faecalis* colonies were counted and multiplied by their dilution factor to get the number of CFU-1/ml.

### 2.9. Antibacterial Activity of the Experimental Irrigants

Five milliliters of the experimental solution was utilized for irrigation of each specimen according to its group and was kept in the canal for 10 min. The needle was inserted to 4 mm from the working length. A final rinse was carried out with 4 ml of sterile physiological saline. A second bacterial sampling (S2) was obtained after the intervention and CFU-2/ml was obtained using the same technique, as previously mentioned.

### 2.10. Statistical Analysis

Data analyses were carried out using Statistical Package for Social Sciences software version 22 (IBM, Armonk, USA). Kruskal–Wallis test was utilized for multiple group comparison, and Student's *t*-test or Mann–Whitney *U* test was used to assess two-group comparison whenever possible. *P*-value < 0.05 was considered a level of significance.

## 3. Results

**Figure 5:**
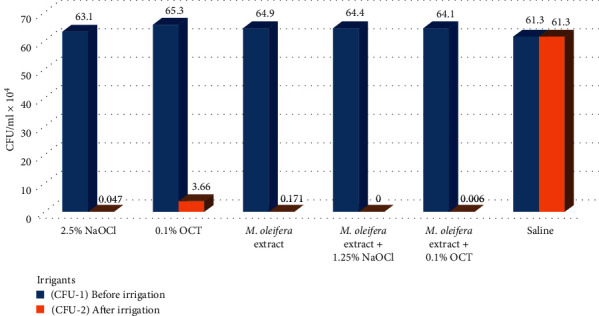
The comparison between CFU/ml of *E. faecalis* in the tested groups before (CFU-1) and after (CFU-2) application of the irrigants.

**Figure 6:**
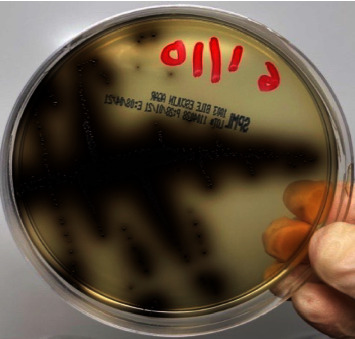
Bile esculin agar showing high numbers of growing colonies of *E. faecalis* before application of irrigants (CFU-1).

**Figure 7:**
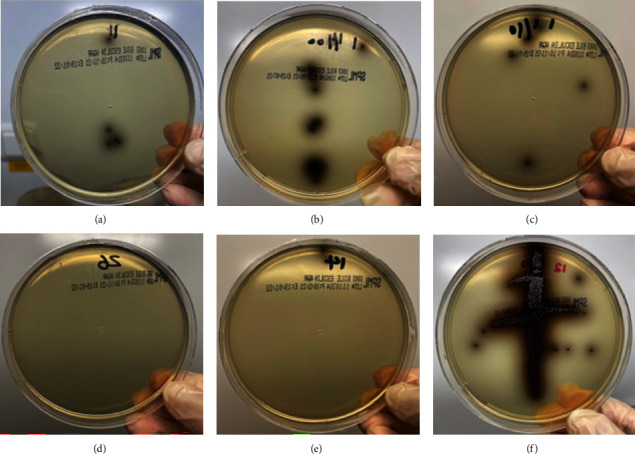
Growing colonies of *E. faecalis* on bile esculin agar plates after application of the tested irrigants (CFU-2): (a) few growing colonies after using 2.5% NaOCl; (b) few growing colonies after using 0.1% OCT; (c) few growing colonies after using *M. oleifera* extract; (d) no growing colonies after using *M. oleifera* extract + 1.25% NaOCl; (e) only one growing colony after using *M. oleifera* + 0.1% OCT; (f) high numbers of growing colonies of *E. faecalis* after using saline.

Comparison between CFU/ml of *E. faecalis* before and after the application of irrigants showed a reduction in the count with a highly significant difference (*P* < 0.001) in all tested groups except in the saline group where the difference was nonsignificant (*P* = 1) (Table 1, Figures 5–7).

**Figure 8:**
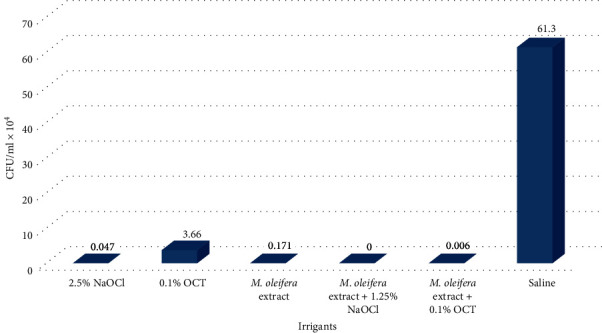
The comparison between CFU/ml of *E. faecalis* in the tested groups after application of the irrigants (CFU-2).

A comparison of *E. faecalis* bacterial counts in all tested groups after irrigation (CFU-2) revealed that the mean difference between the groups was statistically significant (*P* < 0.001). No bacterial count was recorded in group 4 irrigated by the combination of *M. oleifera* extract and 1.25% NaOCl. While the highest mean value of CFU/ml, in the other groups, was noticed in the control group irrigated by saline (61.3 ± 8.367 × 10^4^) (Table 2, Figure 8).

Table 3 shows the comparison between the median and range values of CFU/ml after the application of irrigants of each of the tested groups. A significant difference was reported between group 1 and each of groups 4 and 5, between groups 2 and 4, and between groups 3 and 4. Group 6 has statistically higher CFU/ml than other groups. Other comparisons between groups showed nonsignificant differences.

## 4. Discussion

This study was conducted to investigate the antimicrobial effects of different irrigation solutions that could effectively kill *E. faecalis*. The null hypothesis was rejected as comparison of the CFU/ml in the studied groups showed significant differences.

*E. faecalis* was selected in this study due to its clinical relevance, and previous reporting of its resistance to chemomechanical preparations explained by the thick inner layer of its cell wall termed peptidoglycan which acts as a barrier to many synthetic and natural antibacterial agents [6, 30, 31]. Consequently, the study of antibacterial irrigants for this bacterium is required to improve the prognosis after endodontic treatments [28]. The strain selected was *E. faecalis* (ATCC 29212) as it was used in previous studies [15, 20, 32, 33]. The tested materials used in this study were NaOCl, 0.1% OCT, 100% *M. oleifera* extract, and their combinations, and saline as control.

Sodium hypochlorite (NaOCl) is the most widely used root canal irrigant. Despite the fulfillment of its desirable properties, it has many drawbacks like a cytotoxic effect on the periapical tissues, unpleasant taste, and odor, which increased with increased concentration [10]. Octenidine dihydrochloride (Octenisept) has been proposed as a possible endodontic irrigant due to its antimicrobial activity and low cytotoxicity. Numerous studies showed that OCT has a potent antimicrobial effect against *E. faecalis* [16, 17]. In this study, *M. oleifera* plant was chosen as it has a wide range of antimicrobial properties which have been investigated by some studies, which examined different parts of the plant and different methods of preparation of extract [34].

In this study, *M. oleifera* extract was prepared using ethanol as a solvent because it has hydroxyl groups that can bind polar compounds such as flavonoids and alkaloids components of *M. oleifera* [35]. Peixoto et al. [36] documented that ethanol extracts of *M. oleifera* leaves showed potent antibacterial activity against *E. faecalis* compared to distilled water. The extract was completed using 60% ethanol as a previous study confirmed that the use of 60% ethanol as a solvent extract for plants produces the highest total flavonoid levels compared to ethanol with higher concentrations [37]. These higher flavonoid levels coincide with higher antibacterial effectiveness [38]. In this study, human single-rooted teeth were used instead of disc diffusion methods to imitate the actual environment of root canal therapy. A size 30 file is the bare minimum instrumentation size required to enable irrigant penetration to the apical third of the root canal [39]. Therefore, canals were enlarged using the ProTaper Next rotary system up to size X4 file to guaranty bacterial inoculation and irrigant penetration to the apical third of root canals. All irrigants were left in the canals for 10 min since Berber et al. [40] stated that a contact time of 10 min with NaOCl eradicated the *E. faecalis* strains. A final rinse with sterile saline was used before the second bacterial sampling (S2) [8, 19, 32, 41]. Some authors used neutralizing agents to stop the action of the used irrigants [16]; however, in this study, one of the tested materials was *M. oleifera* leaf extract, an herbal extract without a known neutralizing agent in the available literatures. So, we used saline rinse to standardize the methodology in all tested groups.

According to this study, the number of *E. faecalis* CFU/ml significantly decreased after the application of 0.1% OCT (Group 2). This was in accordance with other studies which confirmed the effect of OCT on *E. faecalis* [16, 17, 41, 42]. Also, we found that 0.1% OCT produced comparable antimicrobial activity to 2.5% NaOCl (Group 1) against *E. faecalis*. These results were in accordance with those of a previous study, which demonstrated that the antimicrobial activity of OCT was comparable to that of NaOCl against *E. faecalis*, *C. albicans*, and a mixture of both using the agar diffusion method [43]. Conversely, Tirali et al. [15] demonstrated that OCT was more efficient than higher concentration of NaOCl (5.25%) for killing *E. faecalis*, *S. aureus*, and *C. albicans* by broth dilution method. Likewise, Anuradha et al. [44] found OCT to be more potent than 5% NaOCl against *E. faecalis* using infected dentin blocks.

As regards to *M. oleifera*, the results of this study showed a significant reduction of *E. faecalis* colony counts after using it as an irrigant (Group 3). This result supports the previous study of Sopandani et al. [29] who proved that 75% and 100% *M. oleifera* extract solutions were effective in killing *E. faecalis* in root canals. Therefore, this study confirmed the antimicrobial potential of this plant.

Meanwhile, our findings proposed that both *M. oleifera* leaf extract and OCT may be useful as an endodontic irrigant. The antimicrobial activity of octenidine could be explained by its ability to interfere with the cell walls and membranes of bacteria [45]. However, the antibacterial activity of *M. oleifera* leaf extract is attributed to its components, such as saponins, flavonoids, tannins, alkaloids, phenolics, and triterpenoids, which have different mechanisms for killing bacteria [26]. Saponins and tannin compounds interfere with the permeability of the bacterial cell wall. However, tannin also causes shrinkage of cell walls and inhibits protein synthesis necessary for its formation leading to bacterial death. Damage of bacterial cell wall and subsequent cell death were also attributed to the effects induced by alkaloids and terpenoid components of the plant where alkaloids interfere with the constituent components of peptidoglycan and terpenoid forms strong polymeric bonds with porin on the outer membrane of the bacterial cell wall. On the other hand, flavonoids inhibit cell membrane synthesis interfering with the metabolism and physiological functions of bacteria by denaturation, coagulation, and subsequent damage of bacterial proteins after forming complex compounds with proteins through hydrogen bonds [29].

The nonsignificant reduction of CFUs in the group irrigated by saline (Group 6) could be explained by the mechanical effect produced by the flow of the solution [46].

However, in combination groups, no growing colonies were noticed in Group 4 (combination of NaOCl and *M. oleifera* leaf extract). A lower concentration of NaOCl (1.25%) was used with *M. oleifera* leaf extract in this group to overcome the potential drawbacks of higher concentrations of NaOCl. On the other hand, the other combination group (combination of 0.1% OCT and *M. oleifera* leaf extract) (Group 5) showed the lowest mean value of CFUs among studied groups. These findings highlighted that the combination of herbal (*M. oleifera*) and chemical agents has a more significant antibacterial effect than herbal or chemical alone. These observations confirmed the results of Al Qarni et al. [32] who found that the lowest mean value of CFU of *E. faecalis* was observed in the group irrigated with a combination of 1.25% NaOCl and neem leaves extract suggesting that the combination of herbal and chemical agents could enhance the antimicrobial effectiveness of both solutions. Similarly, Dutta and Kundabala [33] reported that combining 2.5% NaOCl and neem leaves extract produced better antimicrobial activities than individual irrigants due to the synergistic effect between herbal and chemical agents. Although the lower concentration of NaOCl used, no bacterial count was recorded in the combination group (Group 4). This synergistic activity could be attributed to the previous explanation that combinations of antimicrobial agents or herbal extracts with potential antimicrobial activity targeting the cellular components of a microbe could make more lethal effects than single drug alone [47].

The study limitations involved the use of straight single-rooted teeth with single canal, not representing the variations in teeth morphology encountered in severely curved roots, or roots with multiple canals and multirooted teeth, as isthmuses. Also, the difficulty to retrieve the bacterial specimen from areas other than the main canal such as dentinal tubules. Another important limitation is that it is an in vitro study that does not consider the physiological alterations and biocompatibility of the extract tested. We recommend investigations of the tested materials and their combinations after longer incubation period to ensure mature bacterial biofilm formation. And that future studies focus on testing the effect of combination of chelating agents or acidic solutions, currently used for smear layer removal, with the *M. oleifera* extract and OCT in test tubes to detect any chemical reaction, and in vitro to test the effect of these combinations on the antibacterial effect.

This study suggests alternative root canal irrigants to NaOCl and emphasizes the use of herbal medicine in root canal therapy. Our results proved the synergistic antibacterial effect of plant extracts and chemicals combination, which help to use lower concentration from NaOCl, in root canal irrigation, thus reducing its drawbacks.

## 5. Conclusion

Based on the results and within the limitations of this study, it can be concluded that 100% *M. oleifera* leaves extract and 0.1% OCT solutions have an antibacterial effect against *E. faecalis* and can be used as root canal irrigants. They are as effective as 2.5% NaOCl against *E. faecalis*. The combination of herbal and chemical agents may generate a more potent antimicrobial effect. However, further studies are needed to investigate the biocompatibility and possible toxic effects of the tested irrigants.

## Figures and Tables

**Table 1:** *Enterococcus faecalis* counts before (CFU-1/ml) and after (CFU-2/ml) application of the irrigants.

Irrigants	CFU-1/ml before irrigation (mean ± SD)	CFU-2/ml after irrigation (mean ± SD)	*P*-value
Group 1 (2.5% NaOCl)	(63.1 ± 9.085) × 10^4^	(0.047 ± 0.042) × 10^4^	<0.001
Group 2 (0.1% OCT)	(65.3 ± 11.450) × 10^4^	(3.660 ± 6.769) × 10^4^	<0.001
Group 3 (*M. oleifera* extract)	(64.9 ± 9.993) × 10^4^	(0.171 ± 0.290) × 10^4^	<0.001
Group 4 (*M. oleifera* extract + 1.25% NaOCl)	(64.4 ± 9.845) × 10^4^	0	<0.001
Group 5 (*M. oleifera* extract + 0.1% OCT)	(64.1 ± 6.983) × 10^4^	(0.006 ± 0.0161) × 10^4^	<0.001
Group 6 (0.9% saline)	(61.3 ± 7.365) × 10^4^	(61.3 ± 8.367) × 10^4^	1

**Table 2:** *Enterococcus faecalis* count (CFU-2/ml) of the tested groups after application of the irrigants.

Irrigant	CFU/ml after irrigation (mean ± SD)	*P*-value
Group 1 (2.5% NaOCl)	(0.047 ± 0.042) × 10^4^	<0.001
Group 2 (0.1% OCT)	(3.660 ± 6.769) × 10^4^	
Group 3 (*M. oleifera* extract)	(0.171 ± 0.290) × 10^4^	
Group 4 (*M. oleifera* extract + 1.25% NaOCl)	0	
Group 5 (*M. oleifera* extract + 0.1% OCT)	(0.006 ± 0.0161) × 10^4^	
Group 6 (0.9% saline)	(61.3 ± 8.367) × 10^4^	

**Table 3:** Comparison between the median and range values of CFU/ml in the tested groups after application of the irrigants.

Irrigants	Median (range) ^*∗*^
Group 1 (2.5% NaOCl)	0.05 × 10^4^ (0.0–0.1 × 10^4^)^a^
Group 2 (0.1% OCT)	0.0 (0.0–20 × 10^4^)^ab^
Group 3 (*M. oleifera* extract)	0.005 × 10^4^ (0.0–0.8 × 10^4^)^ac^
Group 4 (*M. oleifera* extract + 1.25% NaOCl)	0.00 (0.00–0.00)^d^
Group 5 (*M. oleifera* extract + 0.1% OCT)	0.00 (0.00–0.05 × 10^4^)^bcd^
Group 6 (0.9% saline)	60 × 10^4^ (53 × 10^4^–72 × 10^4^)^e^

^*∗*^Data labeled with different letters are significantly different from each other (*P* < 0.05).

## Data Availability

Data are available upon request.
